# A Phenomenological Understanding of Aging “Well” With Multiple Sclerosis

**DOI:** 10.1093/geront/gnaf072

**Published:** 2025-02-15

**Authors:** Emma V Richardson, Robert W Motl

**Affiliations:** School of Sport and Exercise Sciences, University of Worcester, Worcester, Worcestershire, UK; Department of Kinesiology and Nutrition, University of Illinois Chicago, Chicago, Illinois, USA

**Keywords:** becoming, being, Heideggarian, hermeneutic, doing

## Abstract

**Background and Objectives:**

As the life expectancy of the multiple sclerosis (MS) community increases, new innovations and understandings of what it is to age “well” are needed. Building on a line of work exploring the meaning and experiences of aging with a disabling condition, and showing how and why people aging with MS experience this phenomenon differently, this paper progresses aging and disability literature by (re)conceptualizing what “wellbeing” means to people aging with MS, and how wellbeing may be enhanced or compromised.

**Research Design and Methods:**

Working with 40 persons with MS over the age of 60, we used a Heideggerian phenomenological framework to co-construct what wellbeing meant among persons aging with MS.

**Results:**

Emphasizing the importance of the “everdayness” of wellbeing experiences, persons aging with MS discussed how wellbeing was related to “doing, being, and becoming; the ability to do the things they wanted to do, be the person they wanted to be, and the autonomy, opportunity, and ability to do something, or become someone, different.” The ability to be, do or become was, however, dependent on, “the power of people,” “sociocultural privilege” and “writing one’s own health narrative.”

**Discussion and Implications:**

These findings, that are contextualized within the sociocultural boundaries of participants’ situations, can help support persons with MS, families and friends, caregivers, health care professionals and interventionists that are working towards enhancing quality of life among persons aging with MS.

Developments within comprehensive multiple sclerosis (MS) care, including pharmacological, rehabilitative, and lifestyle management, have supported an increased life expectancy among those with MS that now matches the non-MS population (Marrie et al., 2015). This so-called “greying” phenomenon (that is, persons with MS living to older age) has seen persons with MS aged 55-65 years become the most populous age demographic in the United States (US), and those in 70s, 80s and 90s age ranges currently more strongly represented than before (Wallin et al., 2019). Such advancements have triggered calls for research to focus on quality of life (QoL) as well as quantity of life (Motl et al., 2018) and, more specifically, (i) what it means to “age well” with MS and, (ii) how this may be facilitated.

A focus on wellbeing among persons aging with MS is not a new venture, and research has highlighted this population generally values spirituality, physical wellness, and psychological health as key components to wellbeing (e.g., McAuley et al, 2007; Simpson et al., 2019). Such components have already become integrated within intervention trials to enhance wellbeing (e.g., Baird et al., 2019), and this highlights the influence and importance of conducting rigorous, high-quality research on wellbeing to enhance the QoL of this group; identified wellbeing components may better inform interventions that shape rehabilitation and healthcare practice for this MS population.

Herein lies our concern. Much previous literature (including those aforementioned) failed to engage thoughtfully in wellbeing theory and philosophy. This is evidenced through conflated, interchanging terminology such as “wellbeing” and “wellness” permeating the literature, and a failure of authors to state which paradigm of wellbeing they aligned to; a common issue in wellbeing literature as a whole (Grénman & Räikkönen, 2015). Further, the methodological designs and lenses chosen by previous authors (e.g., surveys, deductive analysis) do not sufficiently capture the complex nuances, authentic testimonies, and socially bounded experiences that shape what wellbeing is (possible) to individuals in a chaotic, culturally-nuanced, and uncontrollable world. We propose to address these gaps and limitations by crafting a new understanding of wellbeing among persons aging with MS that embraces wellbeing philosophies, and a congruent methodological design.

## Wellbeing Philosophy and Definitions

Defining what “wellbeing” is within a research project is essential as this term has no universal definition (Simons & Baldwin, 2021). In the Global West (where this work was conducted) philosophies of wellbeing tend to revolve around distinctions between hedonic (subjective perceptions of happiness and satisfaction; Keyes et al., 2002) and eudemonic (outcome-based relative to notions of human flourishing; Ryff & Singer, 2008) paradigms (Smith & Reid, 2018). These conceptions have been criticized for not appreciating the passing of time, or the bounded context in which people live (Andrews et al., 2014). Resultantly, others such as Andrews et al., (2014) proposed a feelings-based approach whereby wellbeing emerges through everyday situations and contexts, but this type of understanding too was criticized for a “neoliberal construction of Western individualism” (Mead et al., 2021, p. 1) that did not appreciate a person’s place within an unequal society and subsequent oppressions they may experience (e.g., sexism, racism, ableism, classism, homophobia) that would limit the wellbeing they could experience. Reflecting upon these critiques, we chose to adopt a more phenomenological approach to understand wellbeing in the hope that this embraces new insight, wondering, and questions that can serve the aging with MS population.

Phenomenological lenses of wellbeing may be underpinned by two key central principles; ‘*human-immersion-in-the-world’* and, *“lived obliviousness”* (Seamon, 2018). Human-immersion-in-the-world refers to a recognition that a person cannot be separated from their place in society; economic, cultural, and social (Sarvimäki, 2006). People are *in* their world and shape understandings of experiences such as aging, wellbeing, or MS within the narratives and stories told about such experiences (Seamon, 2018). For example, an individual that has been born into an area of deprivation in the United States will have a different conception of wellbeing than an individual born into affluence in New Zealand. Conceptions that these respective individuals craft will be shaped by the health care and education structures within which they were/are immersed, (un)employment, MS treatment options, the affordability and/or wait times of MS treatment, and societal narratives of aging and MS. By adopting this central phenomenological component, the limitations of previous wellbeing literature regarding a lack of appreciation for context, boundedness, and sociocultural narratives, are addressed.

A second phenomenological concept, *“lived obliviousness,” is contrary to previously mentioned wellbeing conceptions that place central focus and reflections on moments* of wellbeing. Instead, wellbeing is something typically experienced in a person’s “everydayness” (Heidegger, 1962, 2019). Life unfolds, people “get on with it,” and live their lives such that they do not often consciously reflect on being “well” or that their lives may be otherwise (Seamon, 2018); until they are “not well.” This concept of everydayness or *lived obliviousness* gives space for taken for granted notions of living well or QoL to be explored more meaningfully and empoweringly among people aging with MS. That is, just because one has a chronic illness does not limit one’s ability to feel well. Wellbeing is relative, changing, complex, situational, and embodied for each person—not just the absence of disease or illness. To craft a conceptual understanding of wellbeing while aging with MS, we adopted Martin Heidegger’s existential phenomenology.

## Heideggerian Phenomenology

The concept of *Dasein* is central to Heideggerian phenomenology. This term refers to ‘the self’, the person, the entity or *Being* which can ask, “what is being?” (Heidegger, 1962, 2019). It is the foundation upon which Heideggerian philosophy builds its thinking. In the case of this research, Dasein is a person aging with MS. Dasein is embodied. It has a body, experiences the world through its body, feels things in and through its body, does things with its body. This brings Dasein’s body from the background (everydayness, lived obliviousness) to the foreground of experience for this research, and reflections can be made about how this body feels and interacts within Dasein’s bounded and socioculturally situated world.

Dasein and its embodiment cannot be separated from its surroundings, it’s “*Being in the World*” (Heidegger, 1962, 2019). That is, Dasein is “in the world” but the world may be shaped by Dasein, particularly when it comes to ascribing meaning or “truth” to words, norms, or actions. As Inwood (2019) noted, “A word such as ‘hammer’ or ‘culture’ does not have a singular determinate meaning or connotation; it’s meaning varies with the world in which it is used.” (p. 49). As such, exploring what wellbeing is when one ages with MS can be ascribed various meanings by Dasein, and opportunities to appreciate these different meanings are essential for expanding narratives of what it is to be well aging with MS.

A final key concept is that of “*Care*.” Heidegger’s Care has two distinct meanings; to care or worry about something (e.g., aging or MS worsening) and to “take care” of something (a child, person, oneself). These three key concepts, Dasein, Being-in-the-World, and Care are central to Heideggerian phenomenology and the question “what is being?”; “Only if Dasein is care can it dwell in a significant world, and only if it dwells in a significant world can Dasein be care.” (Inwood, 2019, p. 59). Such an understanding can illuminate why, as previous literature has stated, some persons with MS age well and others do not (Richardson & Motl., 2020), and situate this within the grander scheme of wellbeing as a whole. That is, why some people actively engage in behaviors such as exercise, mindfulness, healthy eating etc., to manage aging and MS, whereas others do not.

Heideggerian approaches to craft understandings of wellbeing are numerous (e.g., Sarvimäki, 2006; Seamon, 2018; Todres & Galvin, 2010) and have further been utilized in aging (Lundin et al., 2013) and chronic illness literature (Svenaeus, 2011). This philosophy, therefore, has much to contribute to an understanding of wellbeing among persons aging with MS. The purpose of this work was therefore to explore, “what does it means to ‘age well’ among persons aging with MS in the US?”

## Method and Methodology

Ethical approval was granted by the University of Alabama at Birmingham (IRB-300002531) and all participants gave informed consent.

### Sampling and Participants/Daseins

To recruit participants, the NMSS sent an email blast to U.S. members. Over 300 people expressed interest in this qualitative study by calling or emailing the research center, providing their age, location, MS duration and gender. To manage this amount of interest, as well as ensure meaningful representation of wellbeing among those aging with MS, we used quota sampling and maximum variation techniques. Quota sampling seeks an equal representation from different areas, whereas maximum variation seeks a deliberate range of demographics are purposefully selected (Robinson, 2014). To do quota sampling, the first author created an extensive list of potential participants and divided this list into north, south, east and west, setting the intention of recruiting 10 participants per region (*n* = 40). To purposefully select participants, she then engaged in maximum variation sampling whereby she selected individuals that ensured an extensive variety of age, locations, and disease durations were represented in the data. The first author contacted each person to complete informed consent and a short demographic questionnaire that included participant age, location, gender, MS typology, and years since diagnosis. If a potential participant did not respond or no longer wished to participate, the first author contacted an individual who had the demographics closest to that person. Twenty-nine participants were female, and 11 were male. Age ranged between 60 and 85 years (average = 67.5 years). Disease duration of MS ranged from 3 to 55 years since diagnosis (average = 25 years). Nineteen participants had relapsing-remitting MS, and 21 had progressive MS.

### Data Collection

Data were collected through online semi-structured interviews focusing on participant experiences of aging and wellbeing. Questions involved life history questions as well as specifically open questions about wellbeing. These included; “What gives you joy?,” “What would an ideal wellbeing situation look like?,” “What does wellbeing mean to you?.” The interview guide can be viewed in Supplementary Materials. Interviews ranged between 58 and 118 min with a mean interview length of 78 min. A total of 3,116 min (54.5 hr) of raw interview data were captured which was transcribed verbatim by an external company. The first author checked for accuracy and confidentialized all transcripts through pseudonyms of names and any other potentially identifying details.

### Data Analysis

We engaged in a hermeneutic analysis of data that was inspired by van Manen’s (1997, 2023) “Phenomenology of Practice.” Further detail about this tradition is provided in Supplementary Materials. This was a five stage, interactive, reflective, and cyclical process outlined in detail later.

#### Step one: fore-structuring and theoretical separation

Heidegger stated that a researcher’s ability and reasons for interpreting data in a certain way are based on their previous knowledge and experience of a phenomenon (McConnell-Henry et al., 2009); what he termed “fore-structure.” This embraces that researchers cannot separate themselves from their previous experiences, and must reflect from where their experiences, assumptions, and groundings of a phenomena (such as wellbeing, aging, or MS) comes from, and how these may influence data collection and interpretations (Inwood, 2019). This fore-structure grounds the study’s epistemology (how is knowledge acquired) within the Dasein-researcher relationship and co-construction of what wellbeing is. Explicit fore-structuring conducted by the first author is available in Supplementary Materials

#### Step two: indwelling in language

Heideggerian phenomenology requires researchers to dwell in language and pay attention to what language is saying (or not saying) about a phenomenon (Heidegger, 2019). Thus, to dwell in the data, the first author conducted all the interviews and reread the transcripts for accuracy. She followed van Manen’s (2023) suggestion of focusing on the language of the phenomenon under exploration in a transcript first (by coding, making notes, generating initial descriptions), and thereafter situating that phenomenon in the wider context of the transcript and life of the participant by asking phenomenological questions (step three). The first author (having indwelled in data) identified and highlighted “feeling” words (e.g., joyful, energetic, happy, depressed), embodied experiences of wellbeing, actions and behaviors linked to wellbeing, and stories and anecdotes describing lived experiences of wellbeing.

#### Step three: phenomenological questions on wellbeing

To “get at” the lived experience of participants and the structure with which experiences were crafted (van Manen, 1997), the first author asked the following phenomenological questions of each transcript; (a) What did wellbeing mean to this participant? (b) How was wellbeing experienced in their life? (c) What shaped their experience of wellbeing? Such questions are essential in hermeneutic analysis as these questions shape the structures and essences of experience that help weave the meaning of a phenomenon (van Manen, 2023). Indeed, having explored language in step two, she began to question and write about the locality, structure, narratives, spatiality etc., that linked each person’s conception of wellbeing within their wider world, before building on these with vivid description.

#### Step four: Theming and crafting vivid descriptions of wellbeing phenomenon

In hermeneutic phenomenology the construction of themes are not about identifying patterns within data, but revealing structures of embodied meaning within human experiences (van Manen, 2023). To have phenomenological power, however, the theme must be rich in phenomenological description and vividly describe and reveal nuances of a contextualized experience (van Manen, 1997). Of note, phenomenological descriptions and themes are always one singular interpretation of human experience that will never exhaust possibilities of other ways of being or experiencing a phenomenon (van Manen, 2023). To capture the essence of what wellbeing may be for persons aging with MS, and building upon previous stages, the first author contextualized lived experiences, actions, and structures of wellbeing within a participants’ taken for granted day to day life. Thereafter, she continued writing and editing with reference back to the data until she believed the lived experiences of wellbeing among participants were faithfully captured and would resonate with readers.

#### Step five: “revealing” dasein and (well)being-in-the-aging-MS-world

The final stage was to move back to a Heideggerian lens to explore Dasein’s “Being-in-the-World” with regards to persons aging with MS experiencing wellbeing. The hermeneutic circle was applied whereby the author interpreted the findings through conceptual and theoretical ideas. This is presented in the Discussion section with insights related to empirical, methodological, and practical implications, as the authors wished for focus and deference paid to participants’ testimonies of wellbeing throughout the Results section.

## Results

### “Doing, Being, and Becoming”

Wellbeing among persons aging with MS was defined as “*Doing, Being, and Becoming.” That is, wellbeing when one ages with MS was* “*the ability to do the things they want to do, be the person they want to be, and have the autonomy, opportunity, and ability to do something, or become someone, different.”* Further exploration of the meaning of this wellbeing concept is provided through the rich, detailed testimonies of participants.

#### “Doing”

For many participants, the ability to do what they wanted was integral to wellbeing:

For me, wellness is doing what I want to do. And I may not be able to do it as fast as I used to be able to do it, I may not be able to do it as well as I used to do it, but for the most part, I can kind of do everything I used to do... In one of my really bad times recently, she (*daughter*) goes, “You know, maybe you have to be a grandma now.” And I thought, “No, no. This grandma is going to run the Color Run with her grandson!” (Brenda, 68)

A further repetitive and meaningful word identified across participants’ descriptions of wellbeing was “living”; and this was specifically in the context of living, and learning to live, with MS:

Wellness to me is being able to live with MS. Not die from it, not suffer from it. It’s to live with it and learning how to live well with it. Learning what foods you can eat, what exercises, what vitamins and supplements and if you need medications, what medications…it’s (wellbeing) just learning to live well…Living well for everybody with MS is so completely different…So I think that’s what wellness is for me; knowing yourself, knowing where you fit and where you don’t. (Maggie, 67)

Living “well” with MS was often equated to adopting various lifestyle related activities that targeted traditionally discussed “domains” of wellbeing—in phenomenology this may refer to “Care.” The following testimony provides an example, not an exhaustive list, of various activities which persons aging with MS engaged:

Taking care of yourself, that you’re doing positive things in your life, as opposed to negative things that would impact your life…So, doing things like an exercise routine, whether you go to Pilates, or yoga, or whatever, that you’re doing things to either maintain or improve your flexibility, balance, those kinds of things…And social. I think that’s a piece of it. And I think that you also need to challenge or engage your brain. You can do body stuff, whatever, but if you don’t keep your brain active, or engaged or whatever, whether you read or whether you, whatever, play computer games. But I think your mental piece of it needs to be, you can’t let that slide. (Charlotte, 67)

Participants discussed, however, that wellbeing went beyond traditional domains noted in literature such as mental and physical (e.g., Simpson et al., 2019), towards an overall embodied feeling of contentment that was difficult to name:

Before, I would have thought only physical, but now emotional and spiritual. What more is there? It’s not happiness. Wellness is contentment with body and soul, so physical wellness; doing the best you’re able to, and I guess that goes for everything. The best you got too in compared with what you’re doing, but I’m not so big into extending your life, but just to live your best life that you have. (Laura, 67)

#### “Being”

The ability “to be” was informed and informing of a person’s identity and their desired self:

I am who I want to be; I’m a mom, a grandmom, a church leader, a friend, a wife and for me that is wellbeing. Can I do what I used to? No. But who can when they reach a certain age? It’s not even the MS really, it’s more just the age thing and I am what I want to be at this age. As long as I can still be me, that is wellbeing right there. (Nicole, 82)

Further, “being” was related to embodied feelings of happiness, joy, and independence; “Being happy, joyful, fun to be around, content, the person I would describe myself as and how others would describe me, before MS and now. That’s wellbeing to me – being myself no matter what” (Gill, 63).

Of note, the ability to do and be were underpinned by the belief in evolution, adaption, and adjustment to changing body functionality. Thus, “being,” being “able,” and “doing” were not static points of life but a dynamic and changing journey that one could take ownership of to “become” something new.

#### “Becoming”

An example of “becoming” is movingly explored through testimony by George. George had progressive MS, used a powerchair and was cared for by his wife but provided a key example of “Being-in-the-World” through doing and becoming:

I love reading, I love literature. And appreciation of beauty is a very important, it’s hard to describe. Learning is the most fun of anything. And I have been a big gardener up til now. I can no longer garden. I can’t. I just can’t go out in the heat and garden anymore. However, what I can do is I can learn a lot about gardening. I can have little pots on the back porch and I can learn about things. I can learn about things about the environment. I can read about it...So the gardening that I can’t do there, I can do in my mind…So, that’s something I care about and I can still become something or someone new and improved! (George, 71).

This is an essential finding to support the possibility of being well despite living with chronic illness, and the need to focus on QoL among persons aging with MS. This group, while living in the moment experience aging and/or MS effects, do look to the future and the possibilities life can bring; “striving, that’s me. I’m always striving to be the best I can be socially, physically, psychologically, cognitively. Just keep striving for it. That’s what it (wellbeing) is to me.” (Wilma, 67): “Wellness is all about future. You can’t go back.” (Ursula, 66)

Delving further into the bounded, everyday experiences of wellbeing among persons aging with MS, the ability to embody wellbeing was contingent on three key aspects; (i) the power of people, (ii) sociocultural privilege, and (iii) writing one’s own health narrative.

### The Power of People

Relationships with others, and the quality of those relationships, were central to how participants experienced wellbeing. For example, spouses were lauded for their caregiving and support:

It’s [wellbeing] just learning how to live well and that includes your partner, too…I think the divorce rate in MS is very high and I can see that. But I am so richly blessed. My husband is my biggest supporter. He never brings up the MS in any negative light. He’s the one who said it’s the great equalizer. (Maggie, 67)

Those who were isolated or lacking close family/friends described their wellbeing as very low or non-existent:

I don’t have people. As I said, there’s one woman here in her 90s with her marbles still but that’s it. My cousin is my person and we talk most days but she’s in New Jersey. Neither of us can travel. It’s a lonely existence. I get up, do my crosswords, read my books, play games on the computer, and that’s pretty much it. It’s very isolating…very lonely…I don’t see how I can feel wellness or be well again when I’m stuck here [care home] on my own. (Becky, 65)

The energy or contribution of “positive” people was perceived to strongly influence a person’s wellbeing:

You need to have a good group of friends or whatever it is. And just going to a class is not the same as being with people that you like, or people that are positive. And at the same time, you need to limit your exposure to negative people that suck your energy. I call them black holes…People that are just so negative, and they’re just depressing to be around. And it’s hard to maintain a positive anything, positive even about yourself after a while. It’s like, “Get away from me.” (George, 71)

### SocioCultural Privilege

To appreciate what wellbeing is to a population, it is necessary to understand the context. All participants were based in the United States, and thus tied to the U.S. health care system of insurance through personal means or employment. Participants were cognizant that the potential for wellbeing was mitigated by the quality and access an individual had to insurance. For example, Olivia (66) recognized how insurance through her husband’s employment influenced her ability to “be well” and how the absence of economic freedom or health insurance significantly affected other people with MS:

I recognize we are so fortunate to have my husband’s insurance so I can get my infusion, go to PT [physical therapy], see my neurologist, get the scans. Even with his insurance there are months that are very expensive, but we are very financially comfortable so that isn’t a worry. Without that, I really don’t know what we’d do. It breaks my heart thinking about those with MS that don’t have insurance, especially those that have it so bad they can’t work, so go with no insurance. I’m getting upset thinking about it. It’s evil…If you have money and insurance you can live a hell of a lot better than if you don’t.

This is supported by Paula (65) who at the time of interviewing was forced to choose her health insurance due to retirement:

I have to choose a new insurance as I’m 65 and coz of this disease I have so few options and that’s it, I can’t change my mind. So I’m gonna lose money when I retire, my co-pay is gonna go through the roof, my insurance is gonna suck, so I really don’t know what I’m gonna do. My freedom is gone and I’m gonna be counting cents and dimes to get by.

Relatedly, lifestyle interventions to manage MS symptoms such as nutrition and exercise were widely known amongst the group, but some participants lived in “food poverty” areas where food options were limited, and opportunities for physical activity sparse:

We have a [fresh food store] about 30 miles away and we do a large shop there about once a month. What’s hard though is how expensive fresh, healthy food is. I can’t work no more so it’s just [husband’s] wage so we have to be real careful about what we buy…Cos we are out in the sticks it’s hard for me to be active. I do stuff about the house but I can’t walk outdoors in summer cos it’s so hot and the town doesn’t have a gym or nothing... I go to my neurologist and he tells me eat good, exercise and I know it, but I just can’t see how I can living where we live. (Wilma, 67)

### Writing One’s Own Health Narrative

The contrasting fortunes of participants and how they experienced wellbeing was further exhibited in how they crafted their own narrative of health. We have presented two narratives of making sense of aging with MS where we touched on perceptions of “aging well”; either “Gracefully Conceeding” through a narrative of decline or ‘Kicking and Screaming’ through a resistance narrative (Richardson & Motl., 2021). However, focusing specifically on wellbeing illuminated various different health narratives. First, some participants described themselves as *well and healthy despite MS*:

When people say, “How are you doing? Like, really how are you doing?” I don’t know how to answer that. So, I just say, “Actually, I’m good,” because I am good. I am good, but I do have this capricious, debilitating, incurable disease… I’m mentally in a very good place. Spiritually, I’m good. That’s another way I’m good. Sometimes people will ask me that and I’ll say, “Do you mean mentally, physically, spiritually, economically?” I’ll just go, “Which one of those things do you mean, how I am? So, on all those measures, I’m really good. (George, 71)

What influenced the plot line of narratives (e.g., decline, resistance, wellbeing despite MS) was the direction which they looked in life. Those seeing a future such as George generally told a positive narrative, others such as Robert (60) looked to the past as an anchor and comparison to where he is now:

I used to throw about huge sacks of nails and screws for fun. Never needed to work out or nothing as I played football and wrestled through high school then went into a hard physical job. I don’t know if it was that and MS and age or whatnot that makes me so broken but I look back at pictures of those times and think ‘wow, that is health’. Tall, strong, upright, handsome (laughs). It makes me sad looking back and comparing what I am now but that’s just life isn’t it?

Similarly, there were contrasting perceptions of health and wellbeing linked to ability and maintaining or changing one’s desired activities. Some were open to adapting how they did something that maintained their participation:

Wellness means being able to do everything I want to do. And if I can’t physically manage walking up a hill, climbing a mountain, I’m going to find alternatives. So what are the alternatives to doing the same thing? Just like being an artist. I’m not an artist, but there’s got to be something else I can do. I don’t have the fine motor skills to do knitting, or sewing, so what else can I do? You know? I’m always looking for what can I do, not what I can’t do. So that’s what wellness is. (Sandra, 67)

Others, however, were uncompromising in their ability to do an activity the way they wanted to do it:

I didn’t want to compromise something I was great at. When I couldn’t play (violin) how I wanted I just stopped. It made me too sad to pick it up and see how much I’ve lost…Same goes for painting, when I lose that and can’t paint what I see in my head I’ll stop. If I can’t do what I want how I want, I won’t do it. (Zack, 67)

## Discussion

Quantity of life is increasing among persons with MS, and in a complementary manner QoL must become a key focus for scholars, rehabilitation scientists, and practitioners to ensure this community experiences opportunities for successful aging akin to the non-MS population (Motl et al., 2018). Although previous literature has highlighted the importance of “domains of wellbeing” such as physical, social, spiritual, and psychological (McAuley et al., 2007; Simpson et al., 2019), and these were noted by some participants in this study, wellbeing as a concept and lived experience was much more nuanced and complex. The main contribution to aging and MS literature of our phenomenological definition is its dynamic nature. The action-orientated words of “doing,” “being” and “becoming” personify a dynamic and ongoing engagement to “being well,” and this conception has important implications for scholarship. First, testimonies from participants lend towards embracing a more “feelings-based,” embodied approach to wellbeing (e.g., Andrews et al., 2014) rather than relying on more static, domain-based theoretical lenses such as hedonistic and eudemonic models that have typically underpinned interventions and rehabilitation practice. Reconceptualizing wellbeing as something ‘feeling’ or an embodied phenomena, in this case *the ability to do the things they want to do, be the person they want to be, and have the autonomy, opportunity, and ability to do something, or become, someone different,* depicts wellbeing as an ongoing, empowering, dynamic, fluctuating journey of self and self-discovery that fits within an ever-changing world. Currently, in much MS and aging literature, wellbeing is seen as a “destination,” something to be achieved by ticking the box of various domains, and therein, one will be well. Not achieving these tick boxes perpetuates the fault of not “living well” upon the ill person (a typically medical model perception of disability/chronic illness), which is far too simplistic and discriminatory an understanding to continue within our field. A more complex appreciation of persons aging with MS and their social boundedness, for example, economic freedom, caring responsibilities, and opportunities to engage in wellbeing behaviors is required across rehabilitation sciences, regardless of methodology. Further, this dynamic, action-orientated approach to wellbeing is a more empowering and emancipatory conception where persons aging with MS seek to be well, actively engage in behaviors that improve wellbeing, and explore ways to live life fully, rather than be passive recipients of interventions performed “on” them driven by academics and researchers.

Acknowledging the boundedness of what wellbeing is possible lends to a second important implication for literature and practice. Previous work on wellbeing has been criticized for its reliance on Western neoliberalism (Mead et al., 2021), and its lack of consideration for contextual boundedness. A more phenomenological appreciation of wellbeing addresses this limitation. In our work, the ability to “be well” was discussed by participants within the limits of their social structures, and this highlighted important implications for literature, interventions, and practice that we believe is remiss in past work; persons aging with MS in the United States do not have equal or equitable access to healthcare, resources, food, amenities, information, or relational support to do, be and become what they desire.

A third implication for embracing a phenomenology of wellbeing for aging with MS is the contribution this can make to identifying various oppressions and discriminations that may be experienced in the quest for wellbeing. Rather than a focus on individual domains of wellbeing without context, scholars that choose to address wider social, cultural, and economic barriers in the context of the United States may provide persons with MS the opportunity to engage in wellbeing behaviors that were previously unavailable to them. For example, arrangements/support for healthy, sustainable food preparation in food poverty areas, and distanced-based support for physical activity at home. Further, identifying such social oppressions can ensure the representation of aging and MS within wider discussions around ableism, sexism, racism, and classism currently at the heart of physical activity equity discussions (Hasson et al., 2017).

### Methodological Implications

Previous works that have underpinned wellbeing interventions and practice in aging with MS have often relied on surveys or deductive analyses, and these capture wellbeing in isolation rather than within the complex experiences and lives of people aging with MS. Further, such approaches are not emancipatory in nature as they do not amplify authentic testimonies of persons aging with MS, specify lived contexts, cultural nuances, or bounded limitations of social situations, nor how such experiences influence and are influenced by participants engagement in doing, being and becoming well. Heideggerian phenomenology and its commitment to Dasein as central to experience has much to contribute not only to wellbeing and aging with MS, but living with MS as a whole.

A further concept that can enhance the empowerment of working with persons aging with MS in regard to wellbeing is the Heideggerian concept of Care. Care in this sense is not about receiving care in a passive, one-way direction (e.g., from an intervention, health care provider), but actively making decisions related to caring or worrying about something (e.g., actively making choices to eat well). Care in this phenomenological sense further relates to “taking care” of something, such as a child, parent, or business. This definition is of particular importance for aging with MS. Previous literature has noted the dynamic changing of family roles (Utz, Berg & Butner, 2017) and the difficulty many people with MS experience trying to engage in their own wellbeing when they still bear caring responsibilities to aging parents, spouses, and/or children (Richardson & Motl., 2020). This is an essential consideration going forward as persons aging with MS may also experience being simultaneously cared for and a carer (Richardson & Motl., 2021). Support that addresses a person with MS being able to care for themselves while taking care of others must be considered by health care providers, support groups, and friends and families of persons with MS.

This paper has demonstrated how the tradition of phenomenology may be used effectively to capture *some* experiences of wellbeing, but other traditions may be used pluralistically or individually to explore different aspects of the aging experience. For example, participants in this study discussed the importance of being able to facilitate and experience wellbeing through the ability to narrate their own health stories. This is a powerful finding and one that is supported by other successful aging literature (e.g., Hannum et al., 2017; Phoenix et al., 2011), as well as our previous work highlighting the importance of amplifying affirming narratives of aging with MS such as “aging can make MS better,” and “MS can make aging better” (Richardson & Motl., 2020). There are limited and typically declining plot narratives available to this population, and this can shape how they perceive and make sense of aging (Richardson & Motl., 2021). Utilizing narrative, phenomenology, and other qualitative traditions focused on meaning-making and embodied experience can help expand the narratives and stories about aging and MS that better resonate with this community, and provide narrative paths to aging well with MS.

### Recommendations for Practice and Future Opportunities

We have highlighted the importance of context and social, cultural, and economic influences on how persons with MS experience “aging well,” and it is important to recognize that the context of this work was within the United States. That being said, the United States in itself is a country with diverse cultures, geographic locations, demographics, and climates that impact how persons with MS make meaning from and manage their condition (Richardson et al., 2022), For meaningful impact to support communities aging with MS, health care providers, interventionists and practitioners in the United States should look to the unique culture of the states, perhaps even towns, in which they work. Further, there are disparities in comprehensive care services available in different areas of the United States, and this too must be taken into consideration when designing a model of care for persons aging with MS. In this way, health care and support can be shaped to fit the potentially unique cultures of faith, race, gender, and communities of different parts of the United States, rather than a larger, nationwide model that does not fit within more rural or culturally diverse areas of the country. An important finding that influenced what wellbeing was possible for participants was sociocultural privilege and this is deserving of more rigorous study. A limitation of this work is that we did not capture socioeconomic or racial demographics, nor did we consider a more equitable and inclusive sampling and recruitment strategy to contact those persons with MS in hard-to-reach areas such as areas with internet poverty, little access to healthcare, etc. Future researchers may wish to consider socioeconomic status in their research designs to capture the potential for wellbeing amongst groups that experience more oppression and discrimination than others. A much more diverse exploration of what wellbeing means to persons aging with MS is therefore warranted.

A further rationale for research and interventions to be designed within wider sociocultural-economic spheres is linked to the human experience as shaped by a chaotic, culturally-nuanced and uncontrollable world. Over the last three years, there have been phenomena that have impacted on a global scale (e.g., coronavirus diseasae 2019, economic crises) and these phenomena have affected disabled and chronically ill populations most (Mitra et al., 2023). Thus, the lives and social situations of persons with MS, even in the most “developed” countries, have markedly shifted and may require reinvestigation within the context of this changed world. Researchers and interventionist in countries that have a legacy of research focused on wellbeing experiences among MS populations (e.g., United States, Canada, UK) should revisit their findings and critique how relevant these are in this more uncontrollable, chaotic world, but using a more culturally nuanced lens.

A final recommendation from this work is for global researchers to utilize a similar approach (that is embracing the social, cultural, economic, political, etc., world within which persons with MS live) in their countries. MS literature tends to be written from an ethnographic stance where experiences of MS are saturated from the United Kingdom, United States, and Canada. As noted in this work, social, cultural, and economic boundaries of experiences need to be considered to better appreciate the diverse experiences of aging well with MS, and thereafter create meaningful support for communities in respective areas. Further, there is a dearth of research focusing on more global experiences of persons with MS living beyond Globally North, Western countries (Jacobs et al., 2022). Thus, a cultural, person-centered appreciation of what it means to have MS, age with MS, live with MS, and treat MS from individual countries from the South and East are needed to complement a more Global understanding of disability and chronic illness beyond ethnocentric narratives (Goodley, 2017).

## Conclusion

Utilizing a phenomenological lens, we have highlighted the various ways persons aging with MS engage in wellbeing, and one way in which persons with MS perceive “aging well.” The ability to age well is, however, conducive to the relational support persons with MS receive, sociocultural privilege, and ownership they have overwriting their own health narrative. Future work in the United States and beyond must consider local and wider cultural and social influences that impact how persons aging with MS can experience wellbeing, and work accordingly within the boundaries of those social structures.

## Supplementary Material

gnaf072_suppl_Supplementary_Materials

## Data Availability

The qualitative data are not available for replication in keeping with ethical practices regarding anonymity and confidentiality. The study was not pre-registered.
