# Supplementary material for: A Phenomenological Understanding of Aging “Well” With Multiple Sclerosis
Source: Gerontologist. 2025 Feb 15;65(6):gnaf072. doi: 10.1093/geront/gnaf072 (PMC12105571; doi:10.1093/geront/gnaf072)
Supplement: gnaf072_suppl_Supplementary_Materials [file gnaf072_suppl_supplementary_materials.docx]

**Supplementary Materials**

**Interview Guide**

Questions on Aging and MS

1. Grand Tour: Please tell me about yourself
   1. Age, MS diagnosis, geographic area, job, children, pets, partners
2. Please tell me about your life up until this point
   1. What was your life like before your diagnosis?
3. What impact did an MS diagnosis have on you?
   1. Please tell me about your diagnosis experience.
4. What impact does MS have on you now?
5. How has your MS changed over time?
6. Could you please paint a picture of what your life looks like right now?
7. Describe for me what it’s like having MS and experiencing aging.
   1. What’s changed in your experiences and perceptions from being younger with MS and being older with MS
8. How do you compare to your peers?

Questions on Wellbeing

1. What do you do to feel good?
2. What give you joy?
3. What makes you happy?
4. What helps/ facilitates your ability to do these things that make you happy?
   1. What stops you?
5. Can you tell me a story about when you felt ‘well’
   1. What are you doing? What are you feeling, who is there?
6. What would an ideal wellbeing situation look like?
7. What does wellbeing mean to you?
8. What are key components of wellbeing?
   1. Physical, social, spiritual/ religious – key focus
9. What aspects of life make you feel well?
10. How does wellbeing incorporate into your life?
11. What impacts your wellbeing positively?
12. What impacts your wellbeing negatively?
13. What are your perceptions for the future?
14. Anything I’ve missed/ you would like to add?

**Hermeneutic Analysis**

Hermeneutics is a methodology and approach that aligns with Heidegger’s phenomenological sensibilities in numerous ways. Hermeneutic phenomenological analysis embraces temporality and ‘Being-in-the-World’, in particular, participants’ existence, place and relationships within that world (Sloan & Bowe, 2014). Second, a Heideggerian focus on ‘unconcealment’ or revealing of something (Heidegger, 1962, 2019) is congruent with phenomenological philosophy regarding lived obliviousness and how wellbeing is experienced by this population. The ‘revealing’ of wellbeing - or rather it’s co-constructed creation - will never be neutral, but dependent on the mood of both parties at the time knowledge about the phenomenon are shared (van Manen, 2023). For example, a participant with MS may be having a ‘bad day’ with symptoms causing fatigue and pain that may impact the interview. The testimony they would give on a good day may be very different. This complements Heidegger’s premise of everydayness of experience as we can focus on wellbeing of days that may be good, bad, or indifferent, thereby affording a more nuanced, resonant, and accurate appreciation of wellbeing in the lives of persons aging with MS. Third, hermeneutic phenomenology embraces that the meaning of a phenomenon is stratified, multidimensional, contextual, and individual meaning that it is impossible to capture a single definition (van Manen, 2023). This speaks strongly to ontology. Heidegger spoke deeply about ontology (what is reality?), and posited that phenomenology is an ontological theory whereby situating Dasein as central to what is real allows the researcher to answer questions of what things – like wellbeing – actually are (Svenaeus, 2013) and the many possibilities of what something may be.

**Author Fore structuring**

Important fore-structure for the first author that informed her knowledge and assumptions of MS were crafted over 5-years through conducting qualitative studies with persons with MS, specifically exploring exercise behavior, aging, wellbeing, and interactions with health care providers, as well as being a wellbeing coach for persons with MS for two years. Further important aspects of the first author that could not be separated were her nationality and lived experience of chronic illness. The first author is Scottish and grew up in a social care health system that resulted in narratives and experiences of illness and aging being very different to the US. She had lived in the US for only three years when the data were collected, and still did not perceive she had a strong fore-structure of the US itself. Aspects such as the health care system, different illness narratives, world views on illness, health care and socio-cultural beliefs surrounding aging, wellbeing, and chronic illness were very different to her native country, and something she had to contend with during her interpretations; that is, she had to reflect on the socio-cultural context participants lived in (their Being-and-place-in-the-World) rather than a context with which she was more familiar. Further, at the time of interviews, she was experiencing MS like symptoms that were being assessed for MS, and she shared this (at times) with participants. Specifically, she experienced (through the period of doing interviews, analysis and writing) pain, muscle stiffness and spasms, fatigue, paresthesia, numbness in her legs and arms, and vertigo. She therefore had an insight (though brief) into what living with MS may be like, and has experience living with chronic illness.

**References**

Sloan, A., & Bowe, B. (2014). Phenomenology and hermeneutic phenomenology: The philosophy, the methodologies, and using hermeneutic phenomenology to investigate lecturers’ experiences of curriculum design. *Quality & Quantity*, *48*(3), 1291-1303.
